# The Man of a Thousand Pustules: A Case About Acute Generalized Exanthematous Pustulosis

**DOI:** 10.7759/cureus.32073

**Published:** 2022-11-30

**Authors:** Francisco S Laranjeira, Margarida L Nascimento, Filipa Rocha, Ana M Ferreira, Filipa Malheiro

**Affiliations:** 1 Department of Internal Medicine, Hospital da Luz Lisboa, Lisbon, PRT; 2 Department of Dermatology, Hospital da Luz Lisboa, Lisbon, PRT

**Keywords:** skin biopsy, antibiotics, pustulosis, cutaneous drug reactions, toxidermia

## Abstract

Acute generalized exanthematous pustulosis (AGEP) is a rare entity characterized by fever associated with the sudden appearance of erythematous lesions, on which multiple sterile, non-follicular pustules develop. We describe a case of a 44-year-old healthy male who developed fever and multiple erythematous and edematous lesions with progressive generalization to the entire body, associated with multiple small non-follicular pustules three days after having started flucloxacillin for the treatment of a furuncle. Considering the characteristics of the exanthema, fever, and association with aminopenicillin initiation, AGEP was considered. A skin biopsy revealed subcorneal and superficial epidermal pustules, with foci of spongiosis, papillary edema, and a superficial, perivascular inflammatory cell infiltrate with neutrophils and eosinophils, consistent with the clinical diagnosis of AGEP. The culprit drug was suspended, and prednisolone was started, considering the rash extension, with progressive and complete improvement. Although it is a rare condition, the hypothesis of AGEP should be considered in acute febrile conditions with disseminated pustules. It resolves spontaneously after discontinuation of the offending drug, and the diagnosis is based on clinical presentation and skin biopsy.

## Introduction

Acute generalized exanthematous pustulosis (AGEP) is a rare entity characterized by generalized pustular eruption, usually associated with fever and leukocytosis [1]. About 90% of cases are drug-induced, with antibiotics (penicillins and macrolides) being the most frequently involved. Other drugs can induce this toxidermia, with previous descriptions of the role of calcium channel blockers and antimalarial agents. There are reports associated with viral, bacterial, or parasitic infections and iodine-based intravenous contrast media [2].

It is characterized by the appearance of numerous non-follicular, sterile, small pustules on a background of edematous erythema. Fever above 38°C and leukocytosis with a neutrophil count >7000/µL are usually present [3]. Organ involvement is not common in AGEP but can occur, particularly in older or immunocompromised patients [2]. The diagnosis of AGEP is based on the clinical findings and histologic examination of a skin biopsy. The rapid resolution of the eruption after drug discontinuation also supports the diagnosis.

AGEP is a self-limiting disease with a favorable prognosis, and skin symptoms usually resolve without treatment in one to two weeks after suspending the offending drug. The pustular eruption is followed by desquamation, and courses longer than two weeks are rare. Management also includes supportive care, corticosteroid therapy, and symptomatic treatment of pruritus and skin inflammation [4].

## Case presentation

**Figure 1:**
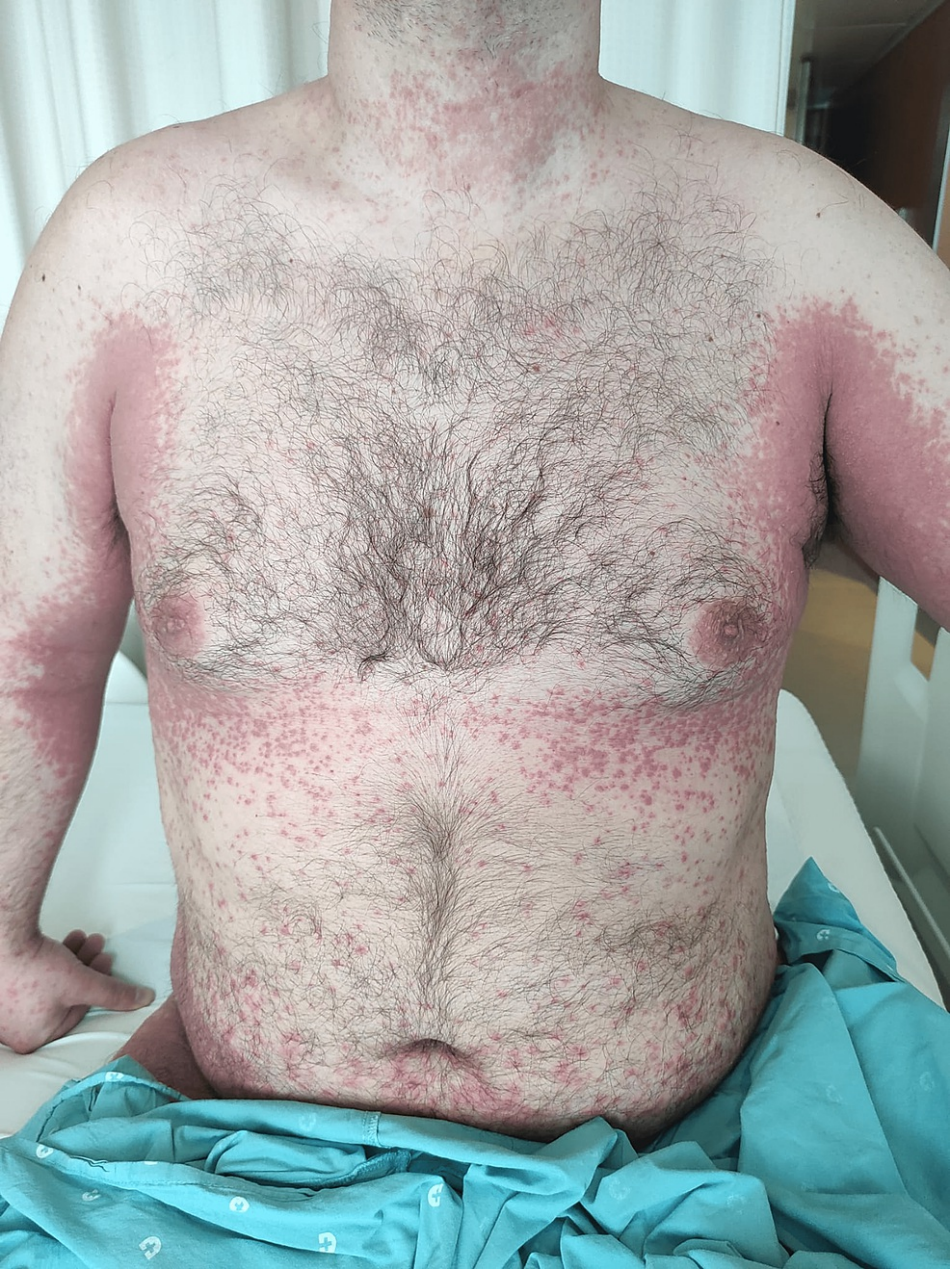
Physical examination on admission to the emergency department

**Figure 2:**
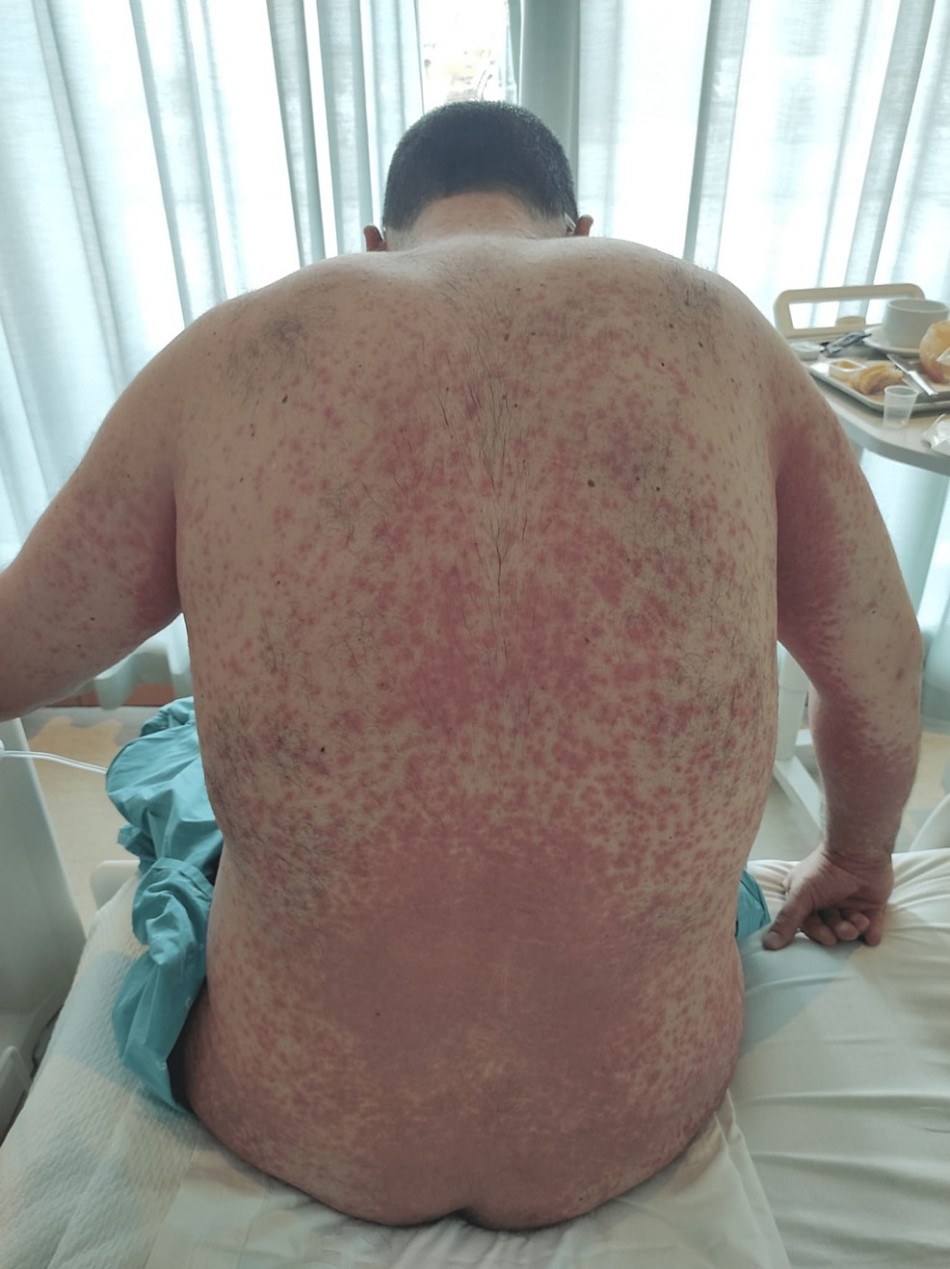
Physical examination on admission to the emergency department

**Figure 3:**
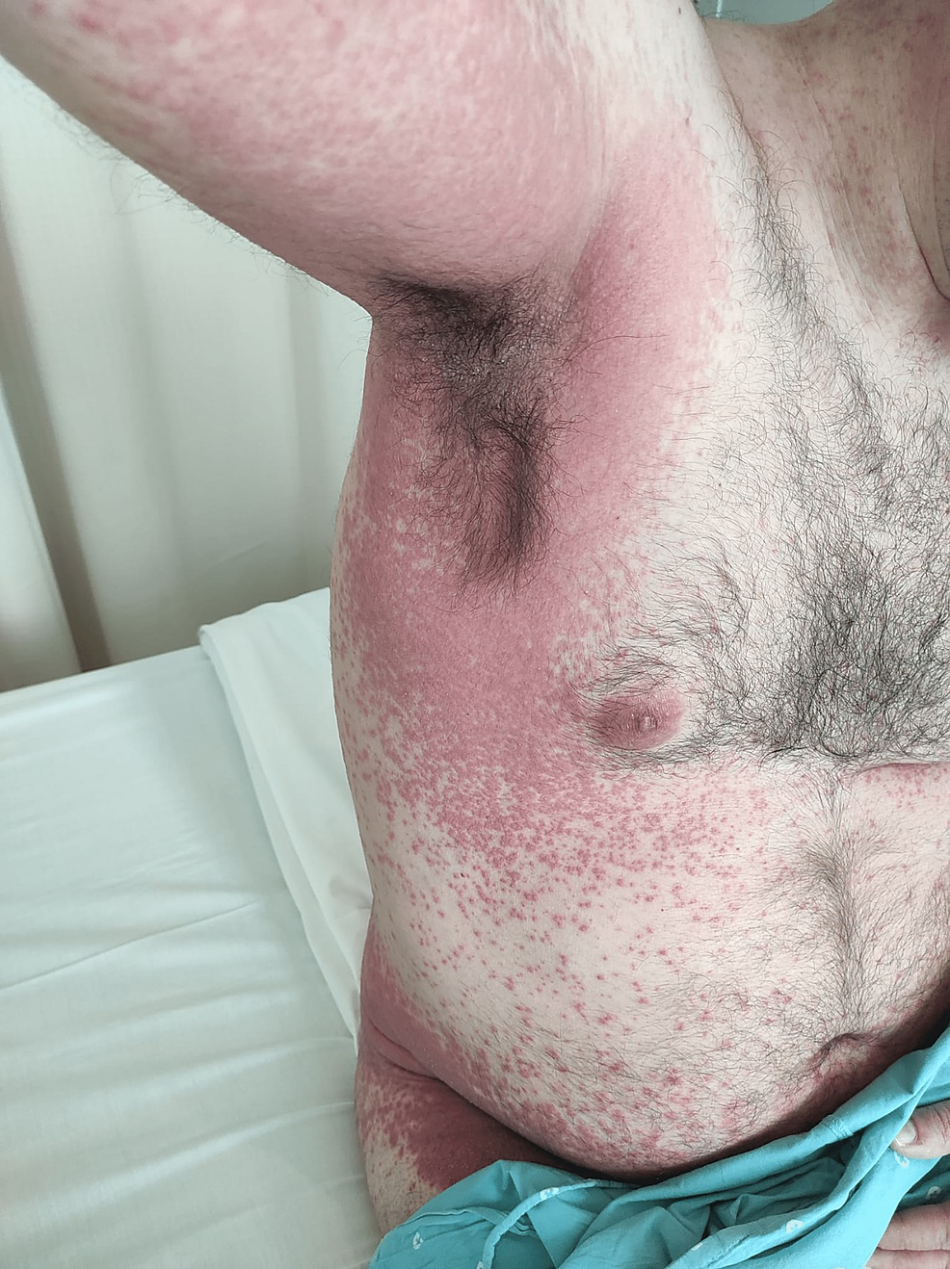
Physical examination on admission to the emergency department: axillary region with confluence of skin lesions

**Figure 4:**
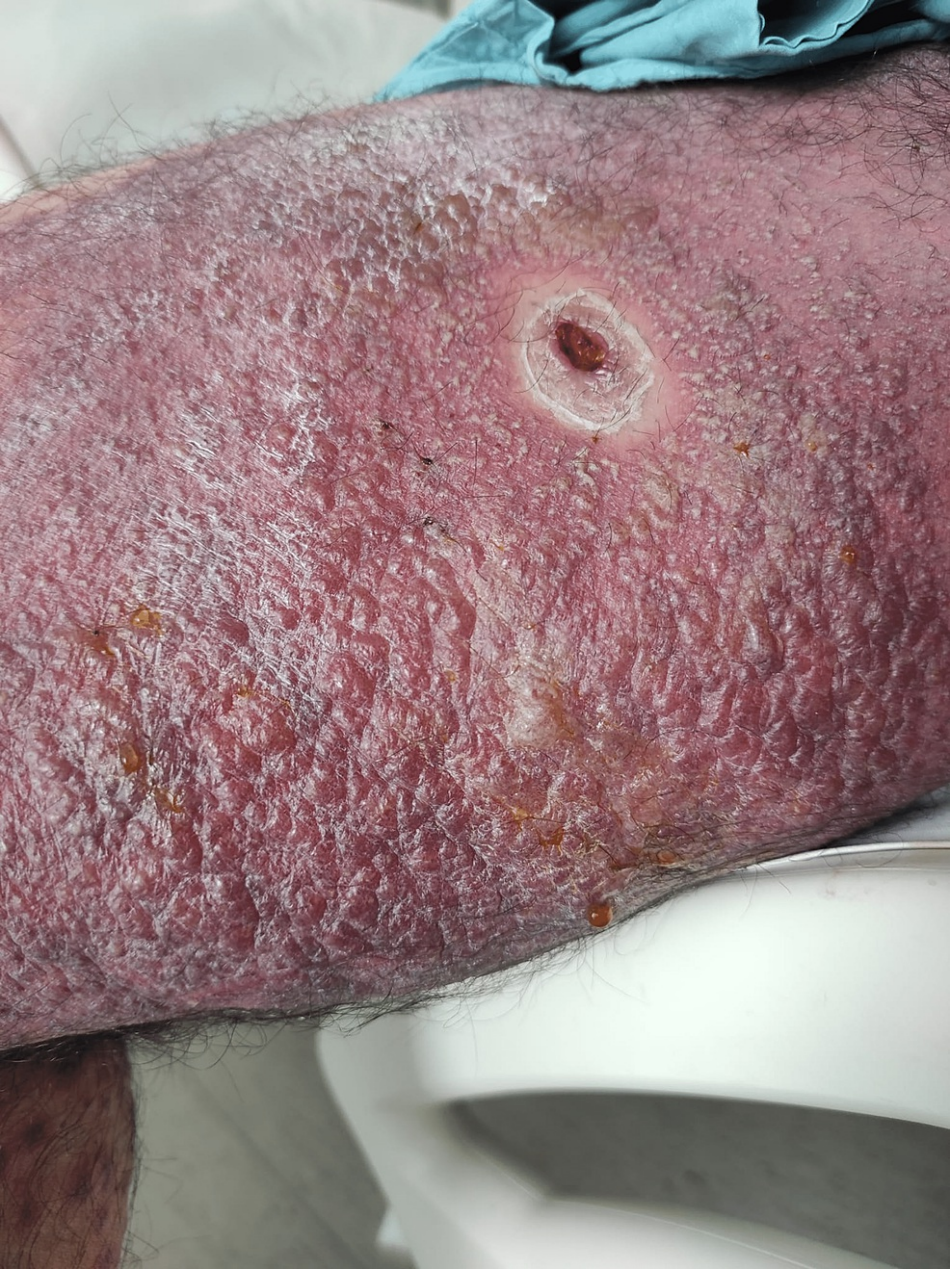
Physical examination on admission to the emergency department: rash sparing the area around the furuncle

**Figure 5:**
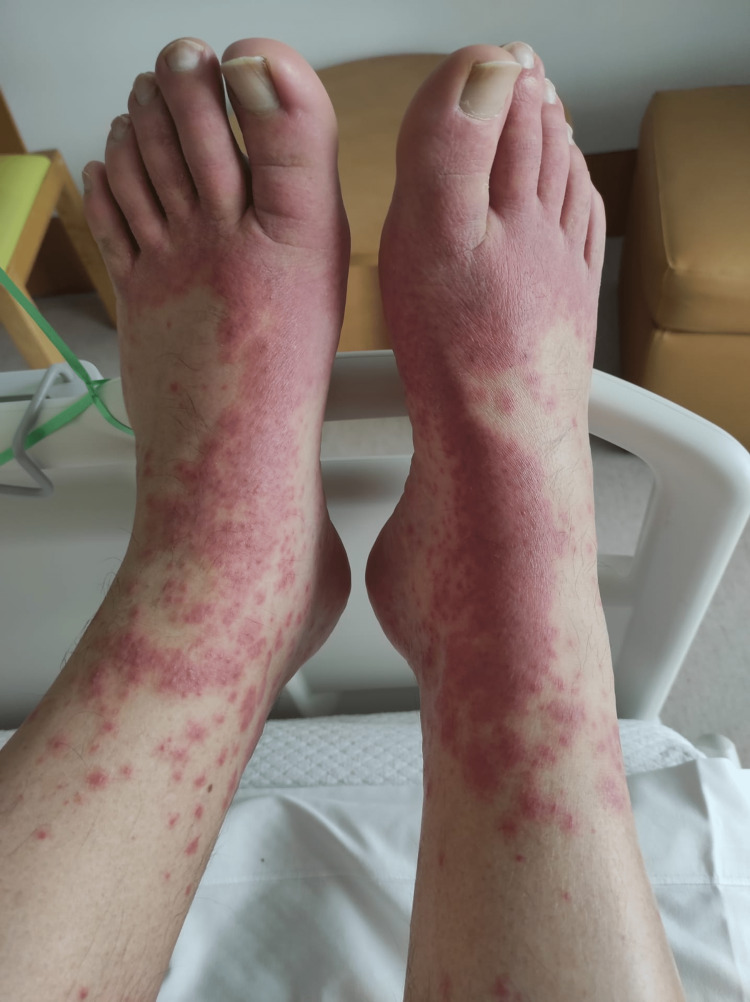
Physical examination on admission to the emergency department: purpura-like lesions on the dorsal region of the feet

A 44-year-old healthy male was admitted to the emergency department with a fever and generalized pustular eruption for the past three days. He had started therapy with flucloxacillin for a furuncle in his left thigh three days before the symptoms appeared. Initially, erythematous, swollen, and pruritic maculopapular lesions appeared. The face, trunk, and proximal area of the limbs were symmetrically affected, with coalescing lesions. In the erythematous areas, multiple small, non-follicular pustules appeared (Figure 1-5). There was a greater exuberance of lesions in the axillary region, as well as enanthem in the palatal mucosa, however, sparing the furuncle area. The skin condition was accompanied by fever (38.3ºC), fatigue, and marked adynamia. Physical examination also revealed purpura-like lesions on the feet and the presence of generalized adenopathies. Complete blood count showed elevated inflammatory parameters (leukocytosis of 15260/µL, 88.4% neutrophils; C-reactive protein of 5.32mg/dL) and acute kidney injury with a creatinine level of 1.40mg/dL.

**Figure 6:**
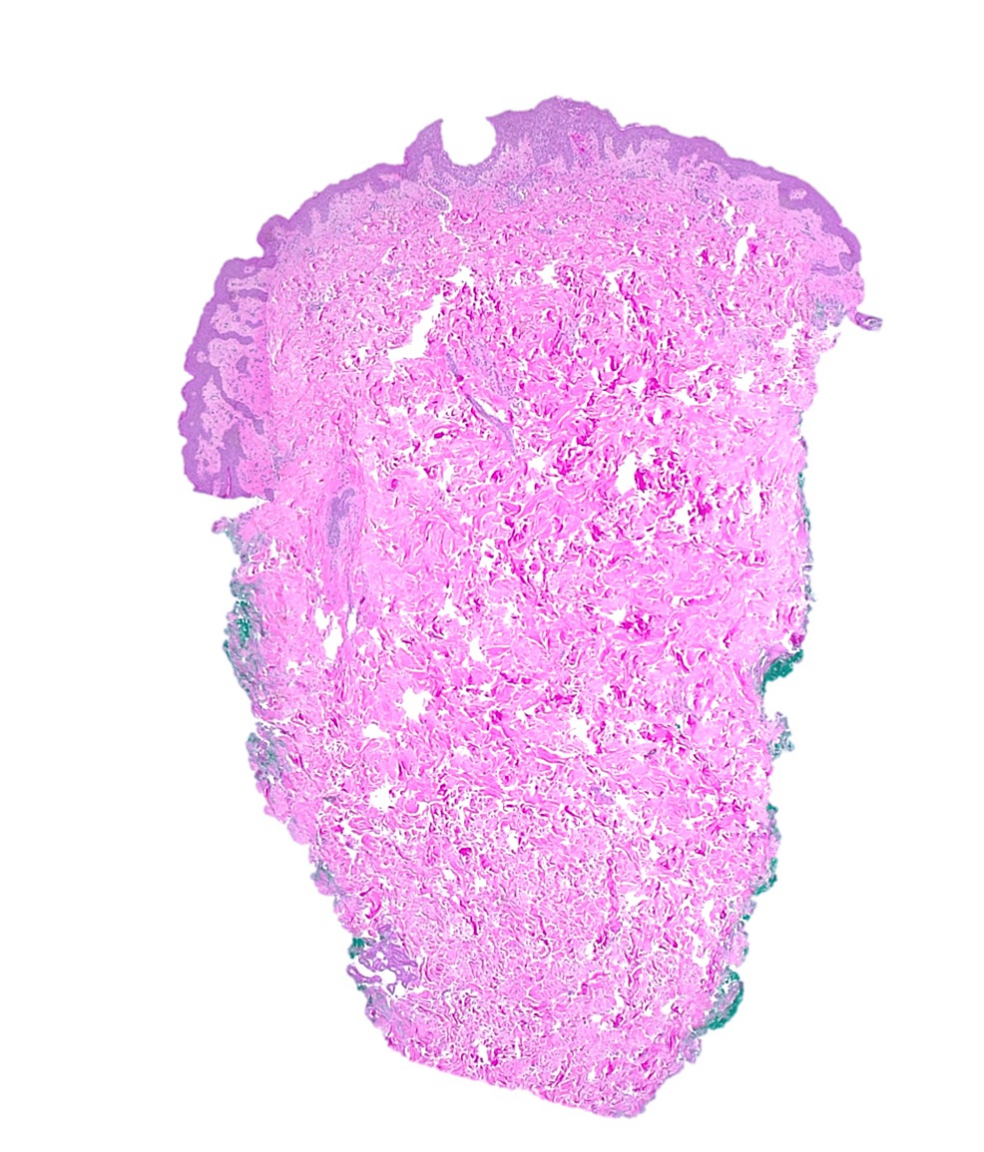
Skin biopsy

**Figure 7:**
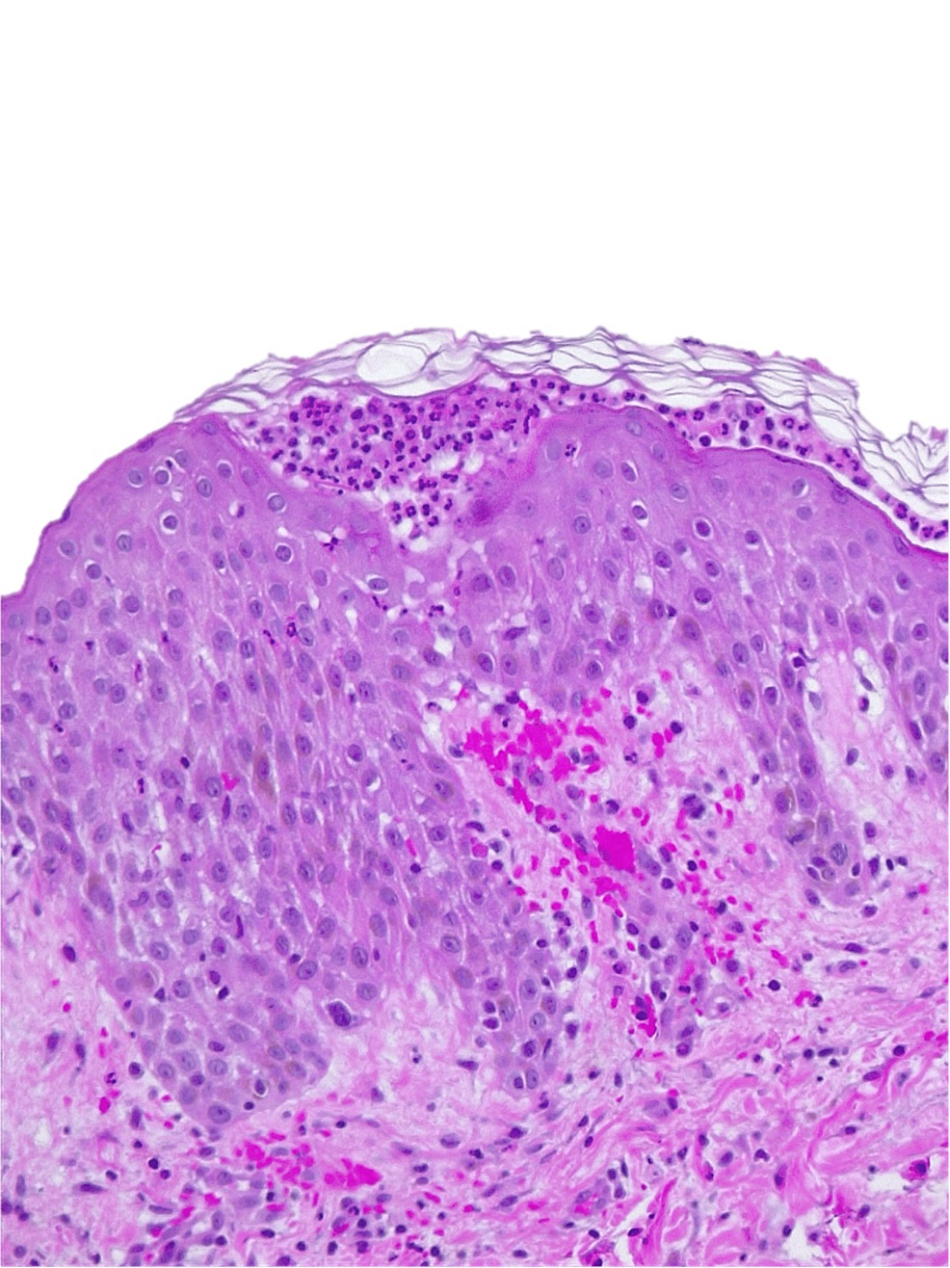
Skin biopsy

Considering the severity of the cutaneous extension, the presence of adenopathies in several chains, and renal dysfunction, hospitalization was decided for an etiological study, clinical surveillance, and treatment. An infectious focus was excluded through clinical, analytical, and imaging evaluation (chest X-ray, blood cultures, urine cultures, pustule content culture, and viral serologies). The patient denied a personal or family history of psoriasis, and given the temporal relationship with aminopenicillin initiation, the characteristics of the exanthema, the presence of fever, and after discussion with dermatology, AGEP was considered. A skin biopsy (Figure 6, 7) revealed subcorneal and superficial epidermal pustules, with foci of spongiosis, papillary edema, and a superficial, perivascular inflammatory cell infiltrate with neutrophils and eosinophils, consistent with the clinical diagnosis of AGEP.

**Figure 8:**
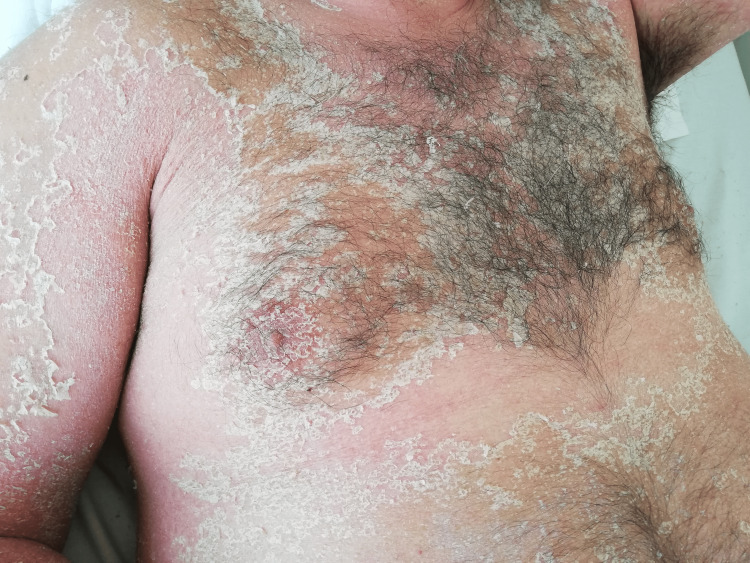
Desquamation phase after starting therapy

**Figure 9:**
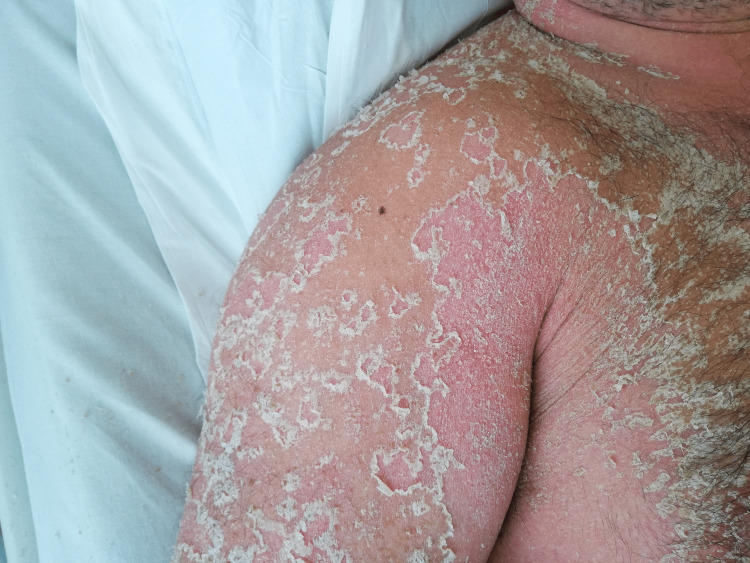
Desquamation phase after starting therapy

**Figure 10:**
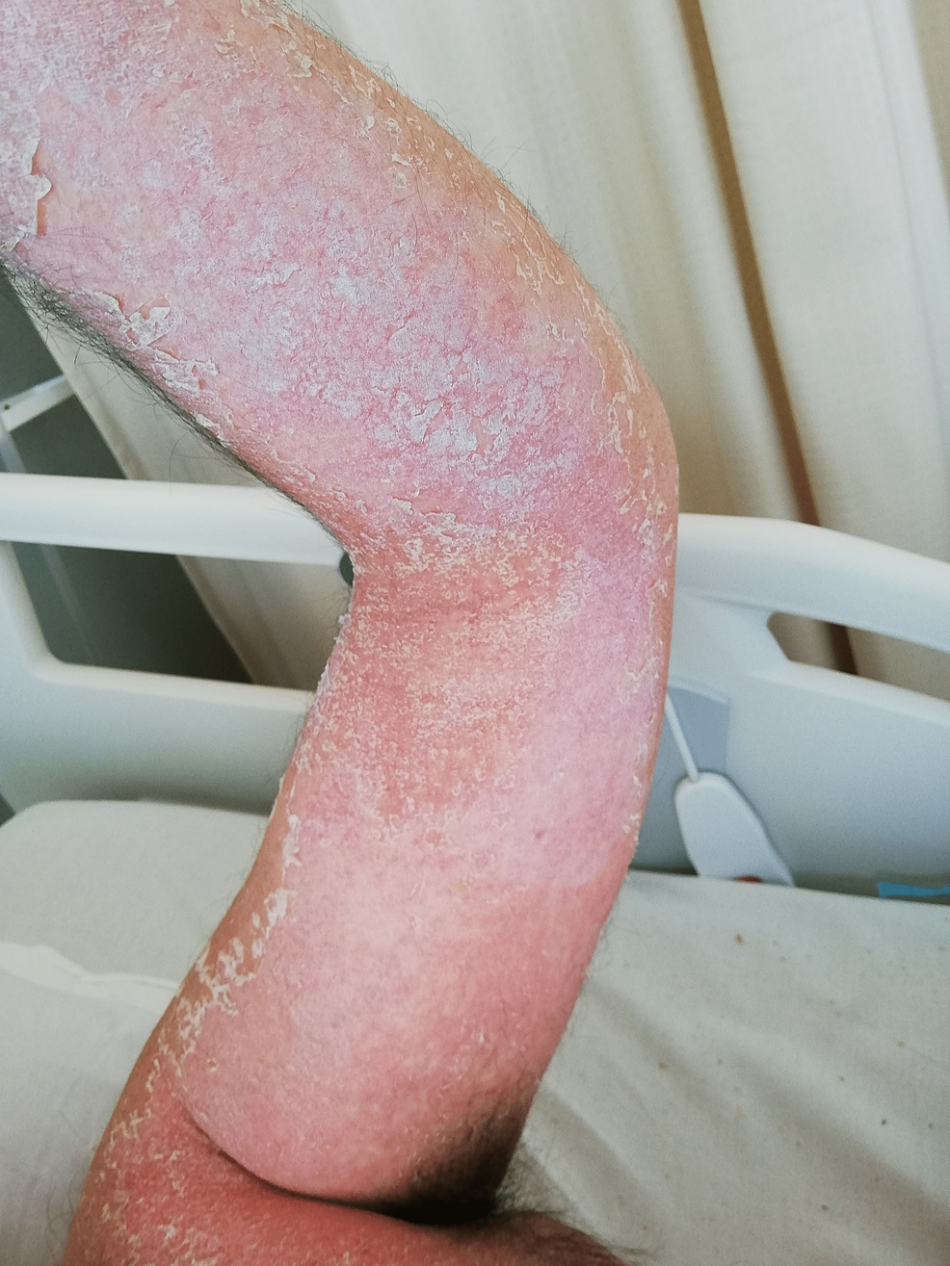
Desquamation phase after starting therapy

The culprit drug was suspended and prednisolone 1mg/kg/day (60mg/day) was started, considering the rash extension, presence of adenopathies and renal dysfunction. For symptomatic relief of pruritus and skin inflammation, antihistamine therapy and topical medium-potency corticosteroids was started (hydrocortisone butyrate emulsion and betamethasone cream). After starting therapy, the patient evolved favorably with a second phase characterized by desquamation of the affected areas with characteristic collarette scaling (Figure 8-10).

**Figure 11:**
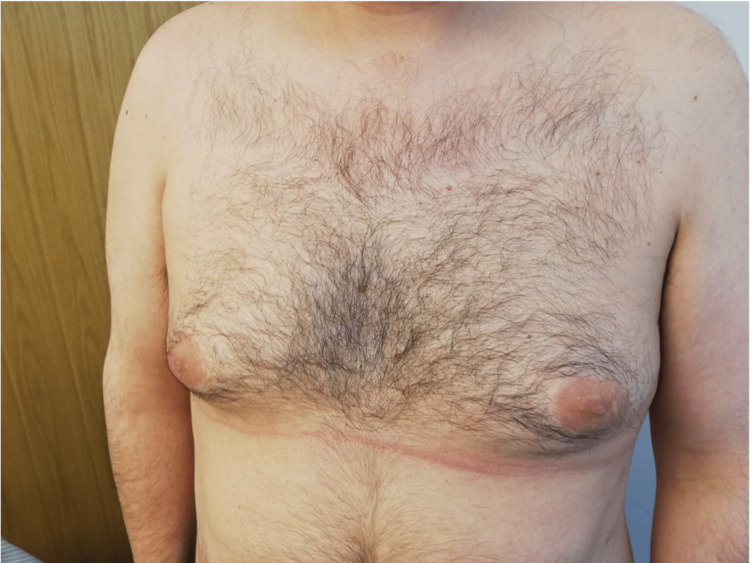
After one month of hospital discharge

**Figure 12:**
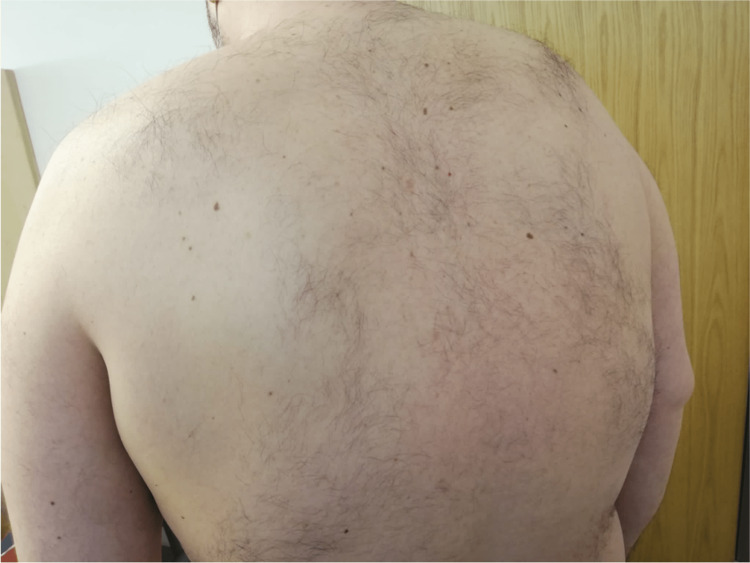
After one month of hospital discharge

**Figure 13:**
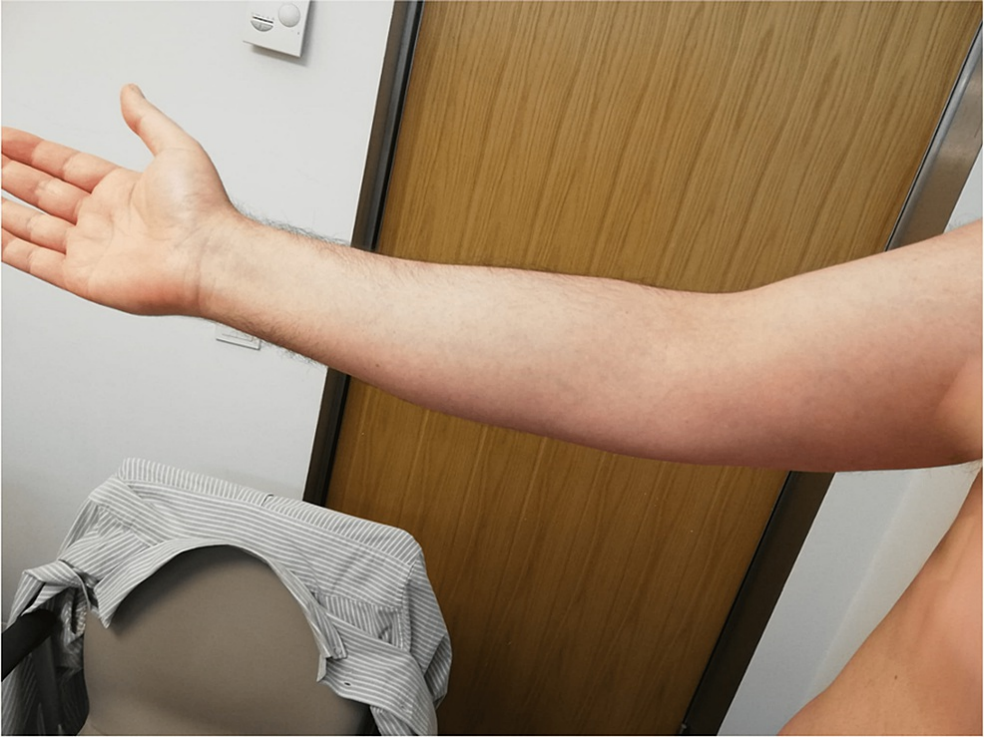
After one month of hospital discharge

**Figure 14:**
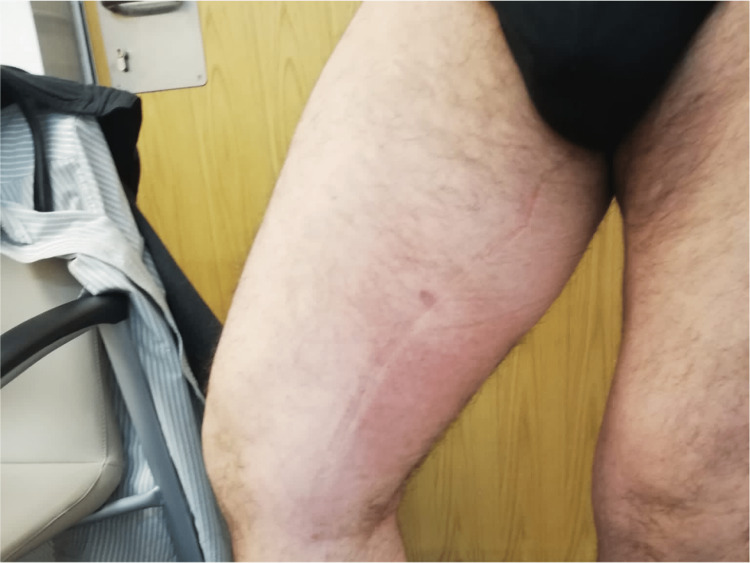
After one month of hospital discharge

Clinical improvement allowed corticosteroid reduction and hospital discharge after 10 days of treatment. Follow-up at one month showed complete resolution of the skin lesions (Figure 11-14).

## Discussion

The term AGEP was introduced by Beylot et al. in 1980 [5] to describe cases of self-limited generalized pustulosis, usually in association with drugs. Antibiotics (penicillins and macrolides) are the most frequently involved, but numerous drugs have been implicated as inducers of this disease [5]. Rarely, AGEP can be induced by non-drug factors, such as contact with mercury, viral infections (cytomegalovirus, parvovirus B19, Coxsackie B4), and recently in association with spider bites [6,7].

In a review of 63 cases [2], the main clinical features of AGEP and their frequency were identified: acute eruption with non-follicular pustules appearing in areas of erythema and edema (100%), fever ≥38ºC (97%), leukocytosis (87%) and neutrophilia (90%). About half of the patients had other skin lesions, including facial swelling, purpura, vesicles, bullae, or target lesions (similar to erythema multiforme). In this study, the mean duration of the pustules was ten days, ranging from four to 30 days. Histologically, it is characterized by the presence of superficial spongiform pustules, papillary edema, and polymorphous perivascular infiltrate with neutrophils and eosinophils. Rarely, there may be areas of leukocytoclastic vasculitis, fibrinoid deposits, and focal keratinocyte necrosis [2]. The AGEP validation score developed by the EuroSCAR study group [8] can be used to help in the diagnosis. This scoring system provides the probability of the diagnosis, and the categories include definite AGEP, probable AGEP, possible AGEP, and doubtful AGEP. Based on this, the diagnosis for our patient was definitive, with a score of 11.

In our patient, the clinical and histological picture was typical, with the disappearance of the pustular lesions 10 days after stopping flucloxacillin. Complete skin normalization occurred at the end of 20 days of treatment, with no residual lesions. The use of oral corticosteroids may have contributed to the rapid resolution of the condition.

Another feature of AGEP is potential organ involvement. In a study of 58 patients, 17% of cases had internal organ involvement, where hepatic, renal, and pulmonary dysfunction were the most common features [8]. Multiple organ dysfunction in AGEP may occasionally require treatment in an intensive care unit. In our case, there was only kidney damage, with normal liver enzymes and no respiratory failure.

The differential diagnosis is essentially made with pustular psoriasis, where inflammatory lesions associated with pustules appear, and should be considered whenever there is a personal or family history of psoriasis, even if limited and stable [6]. The duration of pustular psoriasis is often longer. Rarely, other differential diagnoses such as hypersensitivity syndrome with pustules, subcorneal pustular dermatosis (Sneddon-Wilkinson disease), pustular vasculitis, Stevens-Johnson syndrome (SJS), and toxic epidermal necrolysis (TEN) may be placed [9]. The patient had no history of psoriasis, and the concern for SJS and TEN was low based on the history, lack of significant mucosal involvement, and the close temporal relationship within the start of antibiotics [1].

Treatment consists of identifying and removing the causal agent. The use of oral corticosteroids is not mandatory, but it is often used, given the clinical exuberance of the lesions, in an attempt to induce a faster improvement [2]. Antibiotics are usually not required but may be necessary in case of secondary infection. Mortality is less than 5% in AGEP. When death occurs, it is typically a result of multiple organ dysfunction and disseminated intravascular coagulation [3].

## Conclusions

Although it is a rare condition, this case highlights that hypothesis of AGEP should be considered in acute febrile conditions with disseminated pustules. The diagnosis is based on clinical presentation and skin biopsy, being the brief period of hours to a few days between taking the antibiotic and the onset of symptoms a prominent factor in the diagnosis. Oral corticosteroid therapy is not mandatory because this condition often resolves spontaneously after discontinuation of the offending drug. However, it is often needed, given the clinical exuberance, in an attempt to induce a faster improvement, as this case demonstrates.
